# Metformin promotes ferroptosis and sensitivity to sorafenib in hepatocellular carcinoma cells via ATF4/STAT3

**DOI:** 10.1007/s11033-023-08492-4

**Published:** 2023-06-16

**Authors:** Zongqiang Hu, Yingpeng Zhao, Laibang Li, Jie Jiang, Wang Li, Yuanyi Mang, Yang Gao, Yun Dong, Jiashun Zhu, Chaomin Yang, Jianghua Ran, Li Li, Shengning Zhang

**Affiliations:** Hepato-pancreato-biliary Surgery Department, First People’s Hospital of Kunming City & The Calmette Affiliated Hospital of Kunming Medical University, Kunming, 650032 Yunnan China

**Keywords:** Hepatocellular carcinoma, Metformin, Sorafenib resistance, Ferroptosis, ATF4/STAT3

## Abstract

**Background:**

Hepatocellular carcinoma (HCC) is a common cancer worldwide, and sorafenib is a first-line drug for the treatment of advanced liver cancer. Resistance to sorafenib has become a major challenge in the treatment of hepatocellular carcinoma, however, studies have shown that metformin can promote ferroptosis and sorafenib sensitivity. Therefore, the aim of this study was to investigate the promotion of ferroptosis and sorafenib sensitivity by metformin via ATF4/STAT3 in hepatocellular carcinoma cells.

**Methods:**

Hepatocellular carcinoma cells Huh7 and Hep3B and induced sorafenib resistance (SR) Huh7/SR and Hep3B/SR cells were used as in vitro cell models. Cells were injected subcutaneously to establish a drug-resistant mouse model. CCK-8 was used to detect cell viability and sorafenib IC_50_. Western blotting was used to detect the expression of relevant proteins. BODIPY staining was used to analyze the lipid peroxidation level in cells. A scratch assay was used to detect cell migration. Transwell assays were used to detect cell invasion. Immunofluorescence was used to localize the expression of ATF4 and STAT3.

**Results:**

Metformin promoted ferroptosis in hepatocellular carcinoma cells through ATF4/STAT3, decreased sorafenib IC_50_, increased ROS and lipid peroxidation levels, decreased cell migration and invasion, inhibited the expression of the drug-resistant proteins ABCG2 and P-GP in hepatocellular carcinoma cells, and thus inhibited sorafenib resistance in hepatocellular carcinoma cells. Downregulating ATF4 inhibited the phosphorylated nuclear translocation of STAT3, promoted ferroptosis, and increased the sensitivity of Huh7 cells to sorafenib. Metformin was also shown in animal models to promote ferroptosis and sorafenib sensitivity in vivo via ATF4/STAT3.

**Conclusion:**

Metformin promotes ferroptosis and sensitivity to sorafenib in hepatocellular carcinoma cells via ATF4/STAT3, and it inhibits HCC progression.

**Supplementary Information:**

The online version contains supplementary material available at 10.1007/s11033-023-08492-4.

## Introduction

Hepatocellular carcinoma (HCC) is the fifth most common cancer worldwide, and its incidence continues to rise, with approximately 110,000 deaths from liver cancer in China each year [1]. At present, there is no ideal treatment for HCC [2]. Surgical resection and transplantation are mainly used to treat early-stage liver cancer. Sorafenib, a first-line drug for the treatment of advanced liver cancer, is beneficial for progressive cases or patients who are not suitable for surgery, but many patients with liver cancer respond poorly to sorafenib or develop resistance to it after several months [3, 4]. More appropriate treatments or changes in treatment strategies are therefore needed to achieve better outcomes.

Metformin is a first-line drug for the treatment of type 2 diabetes, and the drug has received attention for its pleiotropic properties and reduced morbidity after treatment [5]. Metformin exerts its antitumor effects by inhibiting the survival and proliferation of cancer cells and inducing cell cycle arrest, autophagy and apoptosis [6–8]. Recent studies have identified potential modulatory effects of metformin on nonapoptotic forms of cell death (including ferroptosis, pyroptosis, and necroptosis) as a potentially promising therapeutic strategy for cancer treatment [9]. Metformin has been shown to exert tumor suppressive effects in liver cancer and other cancers [10]. A number of in vivo and in vitro studies support the clinical use of metformin for the treatment of HCC [11]. Studies have shown that a combination of metformin and sorafenib inhibits cell proliferation and promotes apoptosis, inhibits the epithelial-mesenchymal transition process in vitro and in vivo, and promotes sorafenib sensitivity, which reduces postoperative recurrence and pulmonary metastasis in HCC [12]. In addition, the combination of metformin and sorafenib has been found to enhance the anticancer efficacy of sorafenib by promoting autophagy in HCC cells to induce apoptosis [13]. However, the mechanism of action by which metformin promotes sorafenib sensitivity to inhibit HCC progression is not known.

Ferroptosis is a form of iron-dependent cell death characterized by reactive oxygen species (ROS) generation and iron overload, leading to abnormal accumulation of lethal levels of lipid peroxides [14]. Ferroptosis is closely associated with the development and progression of various diseases, including degenerative diseases, neoplastic diseases, and ischemic injury [15]. The liver is the main site of iron storage in the body, and ferroptosis plays a crucial role in hepatocellular carcinoma. Studies have found that metformin can induce ferroptosis as a potential anticancer agent [16]. Metformin also activates AMPK pathways through the induction of transcription factor 4 (ATF4), which produces anticancer effects [17]. ATF4 is a protective gene for the endoplasmic reticulum and oxidative stress and can activate genes required for various forms of cell stress, thus regulating the adaptive function of cells. Studies have shown that ATF4 affects the antioxidant or pro-oxidation index to influence ferroptosis during HCC [18]. Signal transduction and transcription activator 3 (STAT3) can be activated by ER stress and is involved in regulating ER stress and cell drug resistance [19, 20]. Studies have found that STAT3 knockdown can promote sorafenib-induced ER stress-induced HCC apoptosis [21]. In addition, sorafenib has been found to be a contributor to ferroptosis [22]. The depletion of intracellular iron reserves prevents the induction of oxidative stress by sorafenib in HCC cells, suggesting that ferroptosis may be a target for HCC drug therapy, thereby optimizing the therapeutic effect of sorafenib [23]. The use of metformin to induce ferroptosis may enhance the efficacy of sorafenib, which has important implications for exploring treatment strategies for hepatocellular carcinoma. We speculate that metformin affects hepatocellular carcinoma cells sensitivity to sorafenib through the ATF4/STAT3 molecular pathway.

This study was designed to investigate the promotion of ferroptosis and sorafenib sensitivity by metformin in hepatocellular carcinoma cells via ATF4/STAT3. A metformin-sorafenib drug combination may enhance the therapeutic efficacy of sorafenib in hepatocellular carcinoma, providing a new and promising strategy for the treatment of hepatocellular carcinoma.

## Materials and methods

### Cell culture and processing

The hepatoma cell lines Huh7 and Hep3B were obtained from Procell Life Science & Technology Co., Ltd. (Wuhan, China). Cells were cultured in DMEM (Gibco, USA) containing 10% fetal bovine serum and 1% penicillin/streptomycin in a 37 °C incubator supplemented with 5% CO_2_. Sorafenib (Santa Cruz Biotechnology) was used to establish sorafenib-resistant (SR) Huh7/SR and Hep3B/SR cell models. Cells were transfected at approximately 90% cell fusion using an ATF4 overexpression plasmid (oe-ATF4), siRNA against ATF4 (si-ATF4), and a negative control (oe-NC/si-NC), according to the Lipofectamine 3000 (Thermo Fisher Scientific, USA) instructions.

### Mouse liver cancer model

Tumor cells at the logarithmic growth stage were digested, centrifuged, and placed in DMEM for use. Nude mice (SPF grade thymus-free Balb/C nude mice, male, 4–6 weeks old, 16–18 g body weight) were anesthetized with 3% sodium pentobarbital, and the cells were injected subcutaneously into the right abdomen of the mice. Four weeks later, the mice were euthanised and dissected to observe liver tumor growth.

### CCK-8

Cell proliferation viability was assayed using a CCK-8 kit (Solarbio, Beijing, China). Cell suspensions were inoculated in 96-well plates at a dose of 100 µL per well and incubated at 37 °C for 24 h. Different groups of cells were treated with metformin or sorafenib and incubated for the appropriate times. Ten microliters of CCK-8 solution was added to each well at the corresponding time points and incubated for 2 h. Absorbance at 450 nm was measured using an enzyme marker.

### Western blot

The collected cells were lysed using lysis buffer, and the supernatant was extracted following centrifugation. Protein concentrations were measured using a BCA reaction kit (Thermo Scientific, USA). Target proteins were then separated by 10% SDS polyacrylamide gel electrophoresis, transferred to PVDF membranes and blocked at room temperature for 3 h using 5% skim milk. Prediluted primary antibodies against ABCG2, P-GP, GPX4, ACSL4, ATF4, STAT3, p-STAT3, STAT1, and p-STAT1 (Abcam, UK) were added, and cells were incubated overnight at 4 °C on a shaker with slow shaking, then incubated with the corresponding HRP-coupled secondary antibody for 1 h at room temperature. Immunoreactive bands were detected by the ECL system using an image reader, and densitometric analysis was performed with ImageJ.

### Determination of the Fe^2+^ content

The Fe^2+^ content of the cells was assayed using an iron assay kit (Sigma‒Aldrich, St. Louis, MO, USA) according to the manufacturer’s instructions and as previously described (Wang et al., 2019).

### Determination of ROS content

Logarithmic growth phase cells were collected, digested, and centrifuged, inoculated in 6-well plates, and cultured for 24 h. Different groups of cells were treated, collected after 24 h, and incubated with diluted DCFH-DA at 37 °C in a 5% CO_2_ incubator for 1.5 h. Each group of cells was harvested, and the fluorescence of DCF was detected using flow cytometry.

### Lipid peroxidation assay

Lipid peroxidation levels in cells were analyzed by BODIPY staining. Cells were treated with BODIPY for 30 min, harvested, and resuspended in PBS. The fluorescence of BODIPY was detected using fluorescence microscope.

### Scratch detection of cell migration

Cells were inoculated in 6-well plates and incubated for 24 h before being scribed with a 20 µL gun tip. After scribing was complete, cells were washed 3 times using sterile PBS to remove the scribed cells (so that the remaining gaps were clearly visible to the naked eye), then placed in fresh, serum-free medium and incubated at 37 °C in a 5% CO_2_ incubator. Cells were removed after appropriate time points (0, 6, 12, 24 h), observed under the microscope and photographed.

### Transwell assays for cell invasion

Logarithmic growth phase cells were digested with trypsin and resuspended in serum-free medium. A layer of diluted Matrigel was applied to the upper surface of the PET film, left at 37 °C for 3 h, and dried overnight. Then, 600 µL of medium containing 10% serum was added to the lower chamber, and 100 µL of cell suspension was added to the upper chamber and incubated for 24 h. The lower surface was immersed in 70% methanol solution, fixed for 30 min, stained with crystal violet, microscopically examined, and the number of cells on the lower surface of the PET membrane was counted. The average for the middle and five surrounding fields of view was calculated.

### Immunofluorescence

Cells were fixed using 4% paraformaldehyde for 15 min and washed three times in PBS. Diluted primary antibodies against ATF4 and STAT3 (Abcam, UK) were added dropwise for 30 min at 37 °C, and then cells were washed three times in PBS. Fluorescently labeled goat anti-mouse secondary antibody (Abcam, USA) was added dropwise for 30 min at 37 °C, and the cells were then restained using DAPI (Sigma‒Aldrich, USA). Images were collected with a fluorescence microscope.

### Immunoprecipitation

Cells were collected and lysed using IP lysis buffer, and the supernatant was extracted following centrifugation. ATF4 antibody (Abcam, USA) was added and cells were incubated overnight at 4 °C with slow shaking. Pretreated protein A agarose beads were added and cells were incubated for 3 h at 4 °C with slow shaking to couple the antibody to the protein A agarose beads. The beads were collected by centrifugation and then washed three times with lysis buffer. Immunoprecipitates were analyzed by immunoblotting.

### Immunohistochemistry

Sections were dewaxed in xylene, washed in buffer, and incubated dropwise with diluted primary antibodies against KI-67, ATF4, and STAT3 (Abcam, UK) overnight at 4 °C. The sections were washed in buffer, horseradish-labeled secondary antibody was added dropwise, and the sections were incubated for 2 h at 37 °C. The sections were developed with DAB-H2O2, followed by Mayer hematoxylin staining, washing in water, alcohol fractionation in hydrochloric acid, and dehydration. Neutral gum was used to seal the slices for photography.

### Determination of MDA and GSH content

The levels of the oxidative stress markers MDA and GSH were determined with an MDA assay kit (Beyotime, Shanghai, China) and a GSH assay kit (Solarbio, Beijing, China), according to the manufacturer’s instructions.

### Statistical analysis

All statistical analyses were performed using GraphPad Prism 8.0 (GraphPad Software Inc., San Diego, CA, USA). All experiments were set up in three parallel groups and replicated three times. The results are expressed as the mean ± standard deviation. Differences between groups were compared by t test, and differences between multiple groups were compared by one-way ANOVA. p < 0.05 was considered statistically significant, and p < 0.01 was considered extremely statistically significant.

## Results

### Metformin promotes ferroptosis in hepatocellular carcinoma cells

**Fig. 1:**
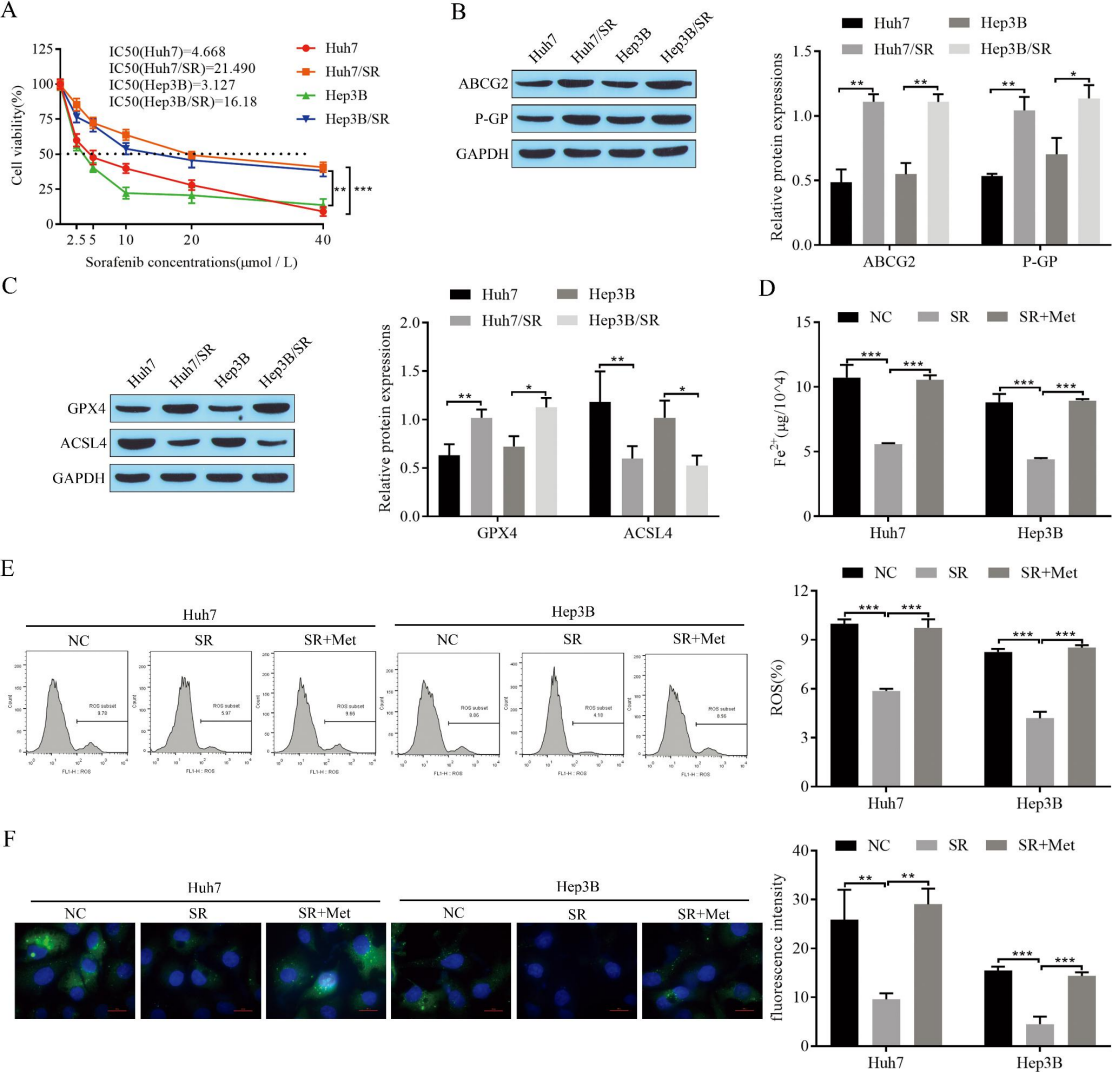
Metformin promotes ferroptosis in hepatocellular carcinoma cells. **(A)** CCK-8 for cell proliferation and sorafenib IC_50_; **(B)** Western blot for expression of drug resistance-related proteins ABCG2 and P-GP in cells; **(C)** Western blot for expression of ferroptosis-related proteins GPX4 and ACSL4; **(D)** Fe^2+^ levels; **(E)** Flow cytometry for ROS; **(F)** BODIPY staining to determine lipid peroxidation levels. *P < 0.05, **P < 0.01, ***P < 0.001

First, the drug-resistant cell lines Huh7/SR and Hep3B/SR were established using sorafenib induction. Huh7 and Hep3B cells were cultured to develop sorafenib resistance by gradually increasing the concentration of sorafenib. The CCK-8 assay results showed a significant increase in sorafenib IC_50_ values in drug-resistant Huh7/SR and Hep3B/SR cells (Fig. 1A). Western blot assays showed high expression of the ABCG2 and P-GP drug resistance-associated proteins in Huh7/SR and Hep3B/SR cells (Fig. 1B). The above results indicated that drug-resistant cells were successfully established. Next, drug-resistant cells were treated with metformin to explore the effect of metformin on ferroptosis in sorafenib-resistant cells. The expression of the ferroptosis-associated protein GPX4 was upregulated and that of ACSL4 was downregulated in Huh7/SR and Hep3B/SR cells, as detected by Western blotting (Fig. 1C). Fe^2+^ levels in drug-resistant cells were decreased and significantly increased after metformin treatment (Fig. 1D). Flow cytometry results showed that ROS levels were reduced in drug-resistant cells and that the addition of metformin significantly promoted ROS production (Fig. 1E). Reduced fluorescence intensity of lipid peroxides in drug-resistant cells was observed by BODIPY staining, while fluorescence intensity was significantly enhanced by metformin treatment (Fig. 1F). The above results suggest that sorafenib-resistant cells inhibit ferroptosis and that treatment with metformin promotes ferroptosis in HCC cells.

### Metformin reverses sorafenib resistance in hepatocellular carcinoma cells

**Fig. 2:**
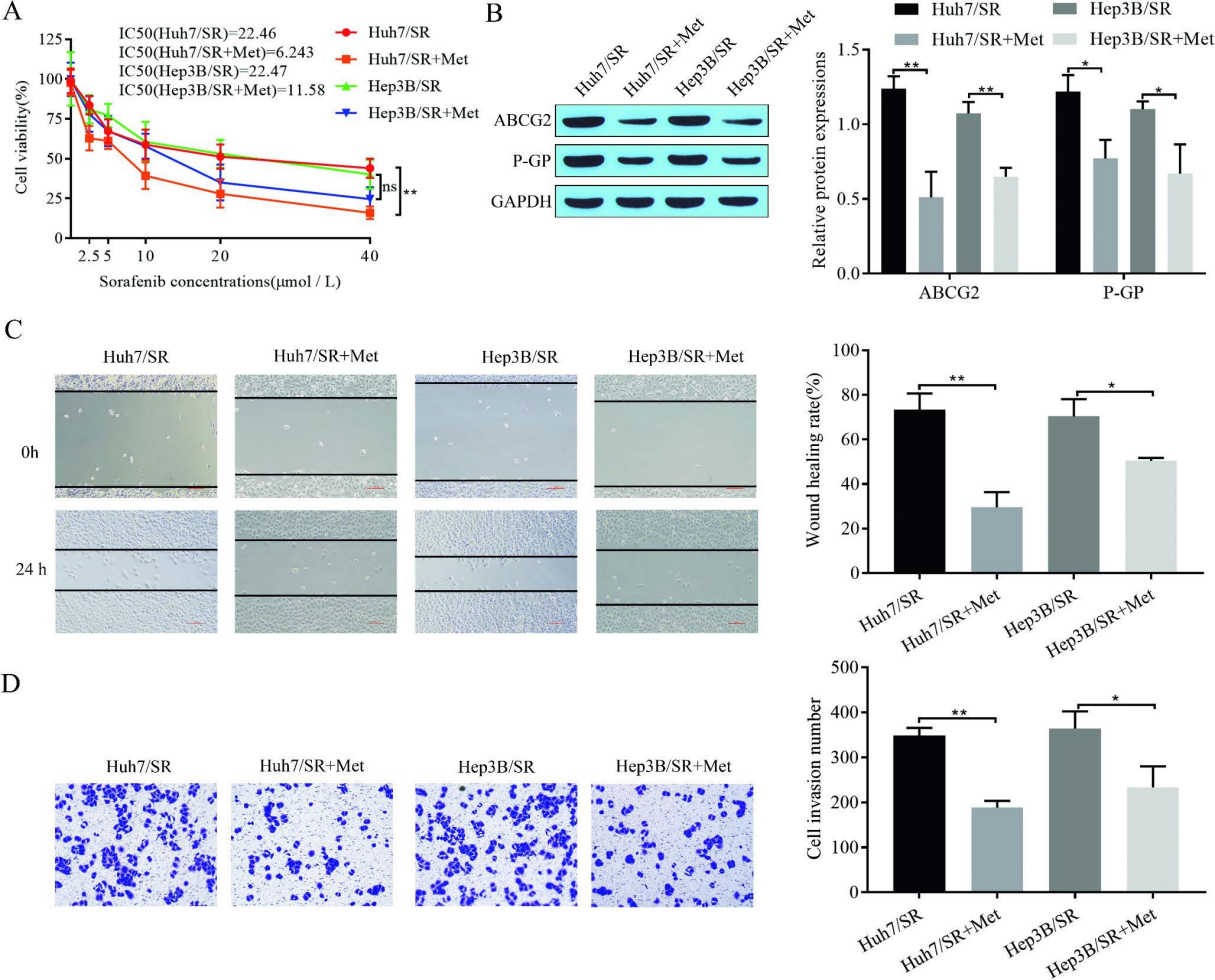
Metformin reverses sorafenib resistance in hepatocellular carcinoma cells. **(A)** CCK-8 for cell proliferation and sorafenib IC_50_; **(B)** Western blot for expression of drug resistance-associated proteins ABCG2 and P-GP in cells; **(C)** Scratch assay for cell migration; **(D)** Transwell assay for cell invasion. *P < 0.05, **P < 0.01, ***P < 0.001

It has been established that metformin promotes ferroptosis in sorafenib-resistant HCC cells. To further investigate the effect of metformin on sorafenib resistance in hepatocellular carcinoma cells, we treated sorafenib-resistant Huh7/SR and Hep3B/SR cells with metformin. The CCK-8 assay showed that compared with the control group, metformin-treated Huh7/SR and Hep3B/SR cells had reduced proliferation and viability, and sorafenib IC_50_ values were significantly lower (Fig. 2A). Western blotting showed that the expression of the drug resistance-associated proteins ABCG2 and P-GP in the cells was significantly reduced after the addition of metformin (Fig. 2B). The results of the scratch assay showed that the migration of drug-resistant cells was significantly reduced after metformin treatment (Fig. 2C). Cell invasion was detected by Transwell assays, and metformin treatment significantly inhibited drug-resistant cell invasion (Fig. 2D). These results indicated that metformin was able to reduce the IC_50_ value of sorafenib, inhibit the resistance of sorafenib-resistant cells, and alleviate cell migration and invasion to reverse sorafenib resistance in hepatocellular carcinoma cells.

**Fig. 3:**
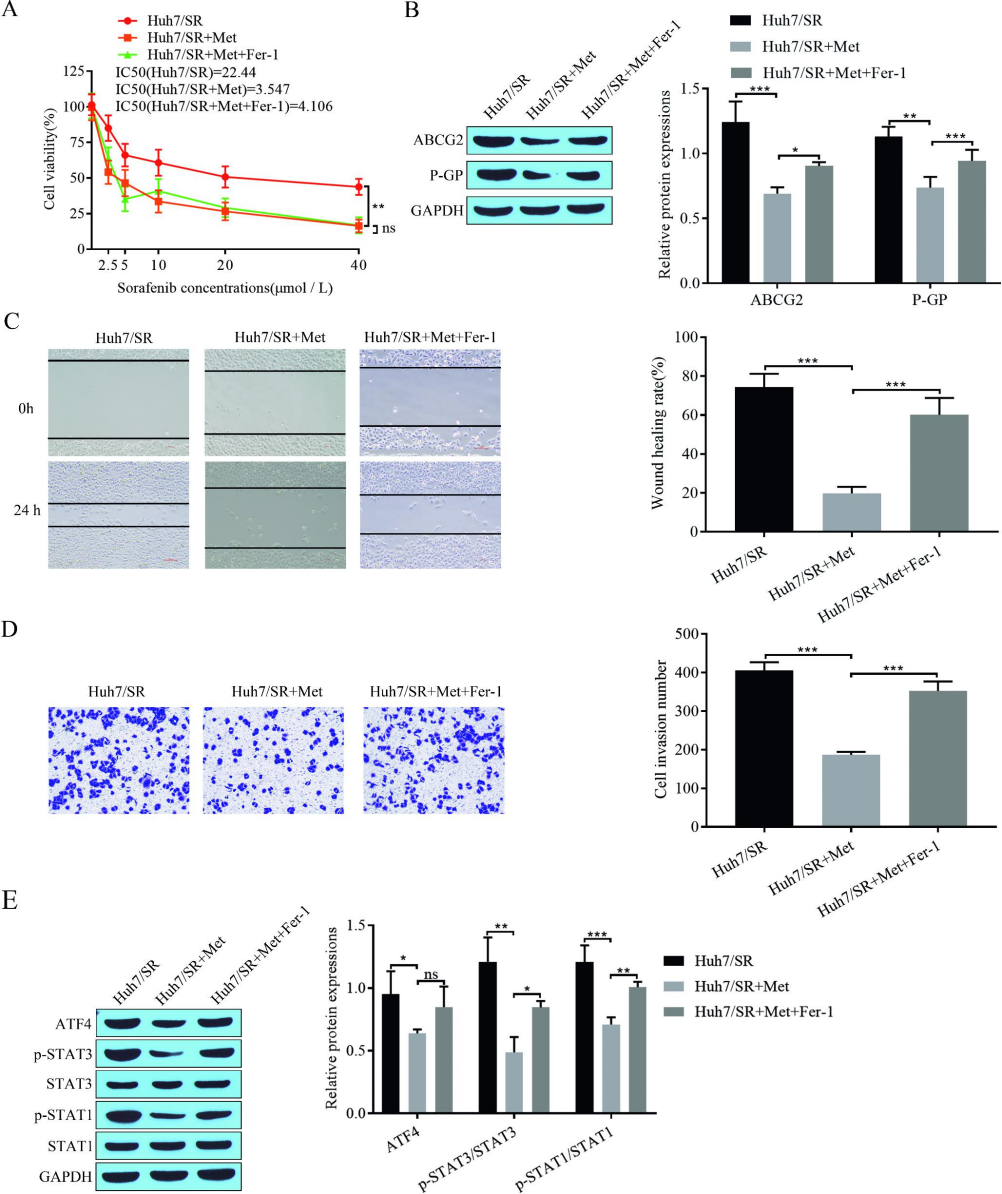
Adding ferroptosis inhibitors weakens the effect of metformin on Huh7 sorafenib resistance. **(A)** CCK-8 for cell proliferation and sorafenib IC_50_; **(B)** Western blot for expression of drug resistance-related proteins ABCG2 and P-GP in cells; **(C)** Scratch assay for cell migration; **(D)** Transwell assay for cell invasion; **(E)** Western blot for expression of signaling pathway-related proteins ATF4, STAT3, p-STAT3, STAT1, and p-STAT1. *P < 0.05, **P < 0.01, ***P < 0.001, ns: no significant difference

### The addition of ferroptosis inhibitors impairs the effect of metformin on Huh7 sorafenib resistance

To investigate the effect of ferroptosis promotion by metformin on sorafenib resistance, we treated sorafenib-resistant Huh7/SR cells with Fer-1, a ferroptosis inhibitor. We determined the cellular value-added viability and IC_50_ of sorafenib by CCK-8, which showed that metformin inhibited cell proliferation and decreased the IC_50_ of sorafenib and that the addition of Fer-1 reversed the effect of metformin and increased the IC_50_ of sorafenib (Fig. 3A). Western blot assays showed that metformin inhibited the expression of the drug resistance-associated proteins ABCG2 and P-GP in the cells, and Fer-1 treatment restored the expression of ABCG2 and P-GP (Fig. 3B). The scratch assay showed that metformin inhibited the migration of drug-resistant cells and that the migration of cells increased after the addition of Fer-1 (Fig. 3C). Inhibition of drug-resistant cell invasion by metformin was detected by Transwell assays, and treatment with Fer-1 reversed the inhibitory effect of metformin and increased cell invasion (Fig. 3D). The expression of the signaling pathway-related proteins ATF4, STAT3, p-STAT3, STAT1, and p-STAT1 was detected by Western blotting, which showed that ATF4, P-STAT3, and P-STAT1 were highly expressed in Huh7/SR cells and that metformin treatment inhibited the expression of P-STAT3 and P-STAT1. The addition of Fer-1 reversed the effect of metformin, and the expression of P-STAT3 and P-STAT1 increased (Fig. 3E). These results suggest that the addition of Fer-1, a ferroptosis inhibitor, can attenuate the effect of metformin on Huh7 sorafenib resistance.

### Metformin alleviates sorafenib resistance via ATF4-induced ferroptosis

**Fig. 4:**
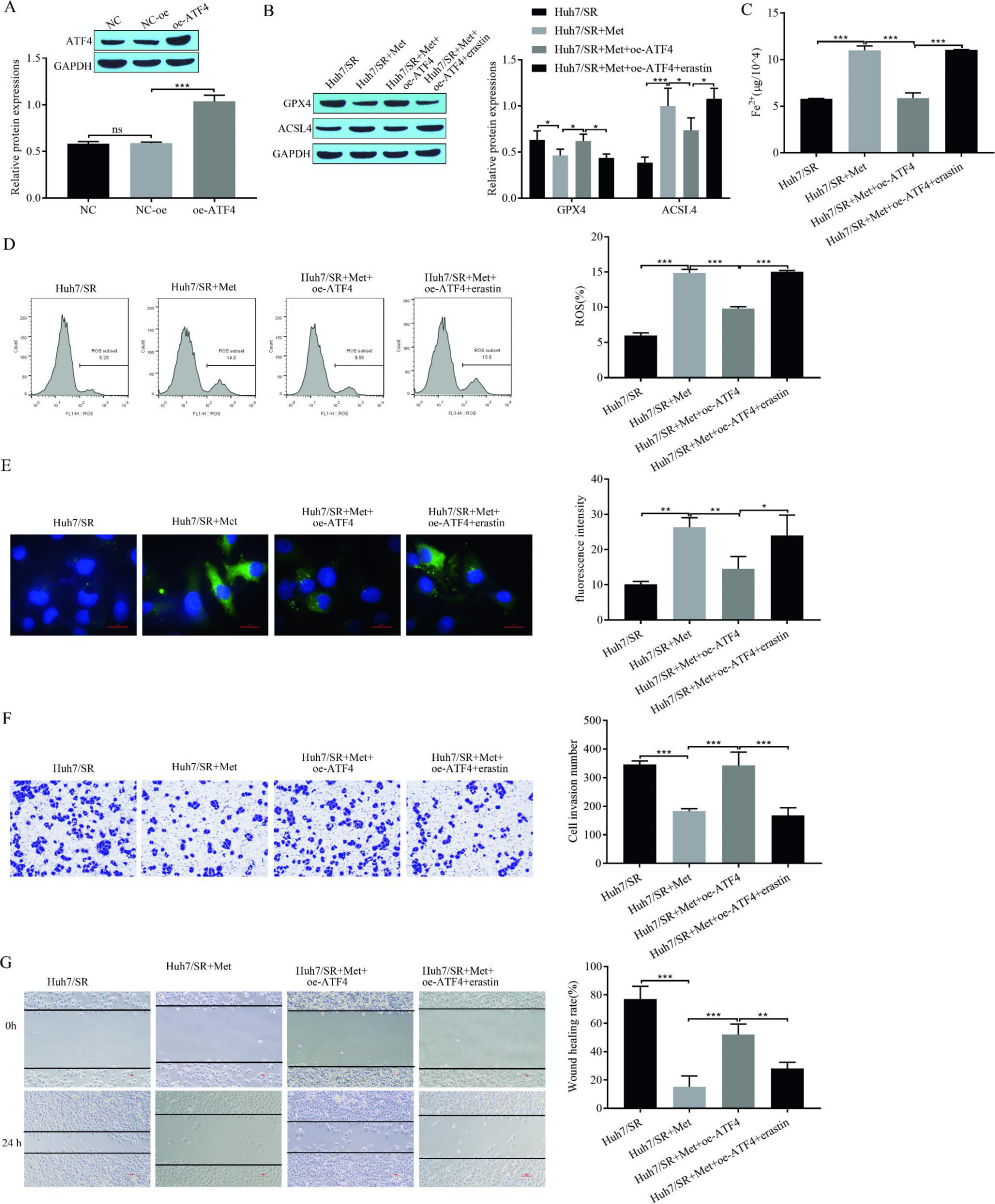
Metformin alleviates sorafenib resistance through ATF4-induced ferroptosis. **(A)** Western blot for transfection efficiency; (B) Western blot for expression of ferroptosis-related proteins GPX4 and ACSL4; **(C)** Fe^2+^ content; **(D)** Flow cytometry for ROS; **(E)** BODIPY staining for lipid peroxidation levels; **(F)** Transwell assay for cell invasion; **(G)** Scratch assay for cell migration. *P < 0.05, **P < 0.01, ***P < 0.001, ns: no significant difference

To further investigate the mechanism by which metformin-induced ferroptosis alleviates sorafenib resistance, we transfected metformin-treated sorafenib-resistant Huh7/SR cells with oe-ATF4 and added the ferroptosis inducer erastin. We detected a significant increase in ATF4 expression in cells transfected with oe-ATF4 (Fig. 4A), indicating that the transfection was successful. Next, Western blotting showed that metformin inhibited the expression of the intracellular ferroptosis-related protein GPX4 and promoted the expression of ACSL4; transfection with oe-ATF4 reversed the effect of metformin, and the expression of GPX4 was upregulated and ACSL4 was downregulated, whereas metformin treatment followed by transfection with oe-ATF4 and the addition of erastin resulted in decreased expression of GPX4 and increased expression of ACSL4 (Fig. 4B). We found that metformin promoted intracellular Fe^2+^ accumulation and that the Fe^2+^ content was reduced in metformin-treated ATF4-overexpressing cells, whereas the Fe^2+^ content was increased again in metformin-treated ATF4-overexpressing and erastin-treated cells (Fig. 4C). Metformin treatment increased intracellular ROS levels as detected by flow cytometry, while ROS levels decreased in transfected oe-ATF4 cells after metformin treatment and increased again in transfected oe-ATF4 and erastin-treated cells after metformin treatment (Fig. 4D). BODIPY staining showed that metformin increased intracellular lipid peroxidation. The level of lipid peroxidation was inhibited by transfection with oe-ATF4, and the level of lipid peroxidation was reduced, while transfection with oe-ATF4 and the addition of erastin after metformin treatment led to another increase in lipid peroxidation levels (Fig. 4E). Transwell cell invasion results showed that metformin inhibited cell invasion. After overexpressing ATF4, cell invasion was increased, while in metformin treatment with overexpression of ATF4 and addition of erastin, cell invasion was reduced again (Fig. 4F). Cell migration was examined by a scratch assay, and the results showed that metformin inhibited cell migration. After overexpressing ATF4, the migration of cells was increased, whereas the migration of cells overexpressing ATF4 and treated with erastin was reduced again (Fig. 4G).These results suggest that metformin promotes ferroptosis and inhibits migration and invasion in drug-resistant cells, whereas overexpression of ATF4 reverses the effects of metformin, and then the addition of erastin restores cellular ferroptosis and inhibits migration and invasion. Thus, metformin alleviates sorafenib resistance by inhibiting ATF4-induced ferroptosis.

### Interaction between ATF4 and STAT3

**Fig. 5:**
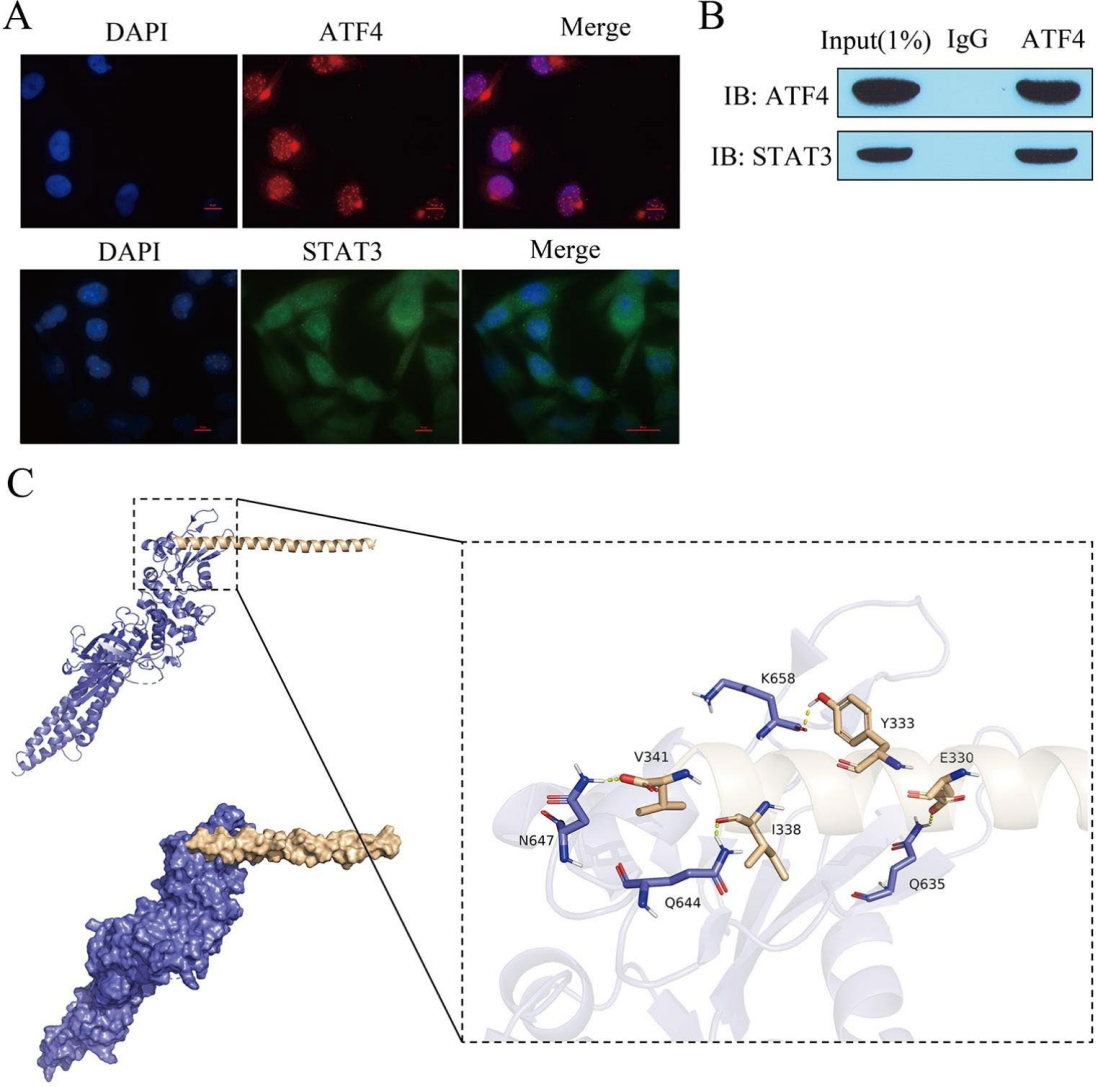
Interaction between ATF4 and STAT3. **(A)** Immunofluorescence localization analysis of ATF4 and STAT3 expression; **(B)** Immunoprecipitation analysis of ATF4-STAT3 interaction; **(C)** ATF4-STAT3 binding force raw signal prediction

By immunofluorescence localization, we found that ATF4 was expressed in the nucleus, while STAT3 was expressed in the cytoplasm (Fig. 5A), suggesting that the regulatory interaction between ATF4 and STAT3 does not occur at the transcriptional level. Immunoprecipitation results showed that enrichment of STAT3 was detected in the ATF4 antibody pull-down complex (Fig. 5B), suggesting a protein interaction between ATF4 and STAT3. The above results suggested that the interaction between ATF4 and STAT3 was likely to have occurred with nuclear translocation. Next, we used ZDOCK 3.0.2 to perform protein binding raw signal prediction for ATF4 with STAT3 and obtained the mode of interaction of ATF4 with the STAT3 protein. The ATF4 protein is shown in beige and the STAT3 protein is shown in blue. The ATF4 protein is seen in the cartoon diagram interacting with the active structural domain SH2 in the head of the STAT3 protein as a beta-sheet interacting with the helix, indicating that ATF4 may prevent SH2 from undergoing phosphorylation. As can be observed from the interaction on the left, V341, I338, E330, and Y333 on the ATF4 protein form hydrogen bonds with N64, Q644, Q635, and K658 on the STAT3 protein (Fig. 5C).

### Metformin mediates ferroptosis and sorafenib resistance via ATF4/STAT3

**Fig. 6:**
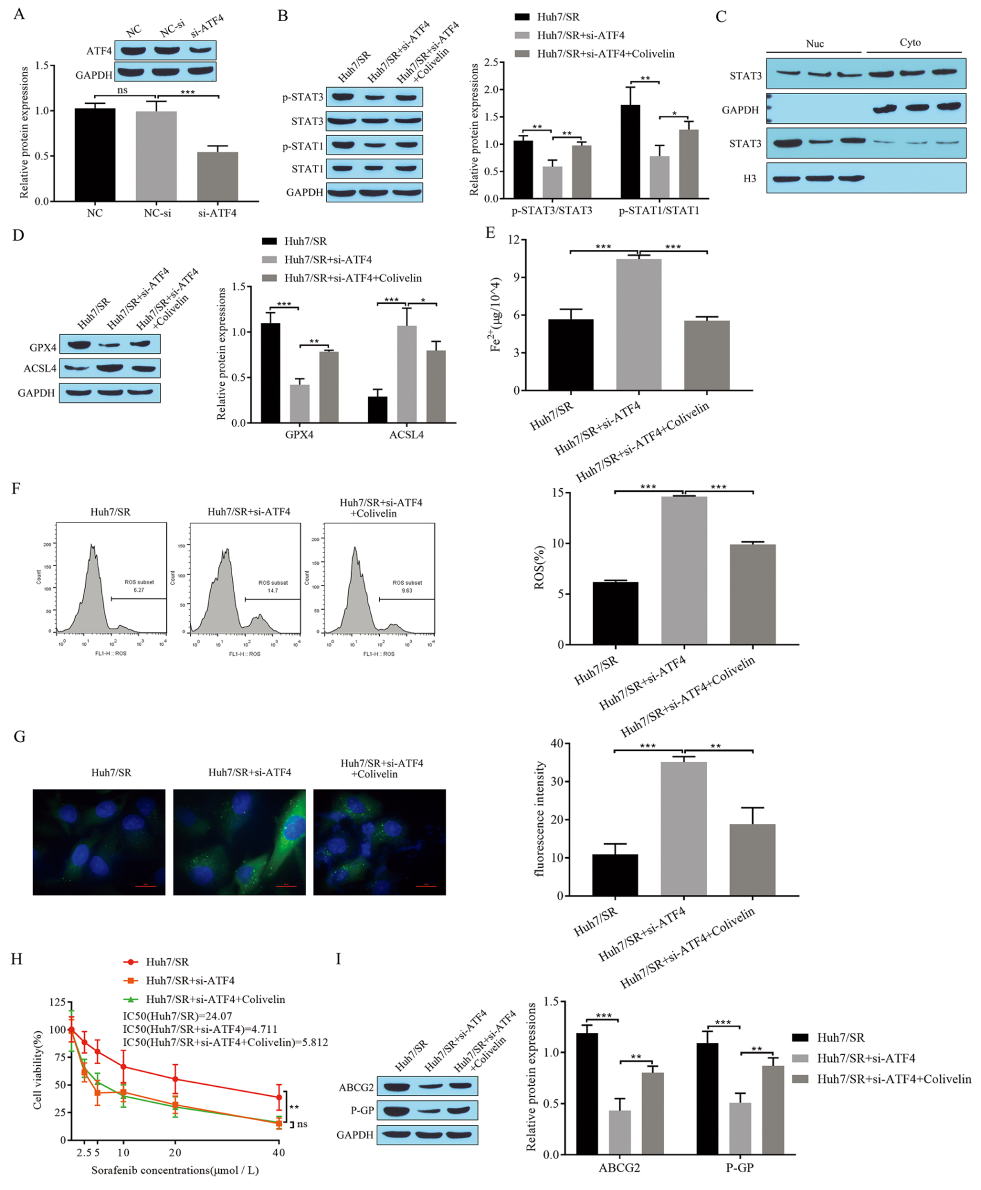
Metformin mediates ferroptosis and sorafenib resistance via ATF4/STAT3. **(A)** Western blot for transfection efficiency; **(B)** Western blot for expression of signal pathway-related proteins STAT3, p-STAT3, STAT1, and p-STAT1; **(C)** Western blot for STAT3 phosphorylated nuclear translocation; **(D)** Western blot for expression of ferroptosis-related proteins GPX4 and ACSL4; **(E)** Fe^2+^ levels; **(F)** Flow cytometry for ROS; **(G)** BODIPY staining for lipid peroxidation levels; **(H)** CCK-8 to detect cell proliferation and sorafenib IC_50_; **(I)** Western blot to detect expression of drug resistance-associated proteins ABCG2 and P-GP in cells. *P < 0.05, **P < 0.01, ***P < 0.001

Metformin alleviates sorafenib resistance through ATF4-induced ferroptosis, and a further interaction between ATF4 and STAT3 has been demonstrated. Therefore, to investigate metformin-mediated ferroptosis via ATF4/STAT3 in sorafenib resistance, we transfected sorafenib-resistant Huh7/SR cells with si-ATF4 and added the STAT3 activator Colivelin. We detected a significant reduction in ATF4 expression in cells transfected with si-ATF4 (Fig. 6A), indicating that si-ATF4 transfection was successful. The expression of the signaling pathway-related proteins STAT3, p-STAT3, STAT1, and p-STAT1 was detected by Western blotting, and the results showed that the expression of p-STAT3 and p-STAT1 was significantly reduced after transfection with si-ATF4, and the expression of p-STAT3 and p-STAT1 was increased again after transfection with si-ATF4 and the addition of Colivelin, whereas STAT3 and STAT1 did not change significantly (Fig. 6B). The results of the Western blot assay showed that transfection with si-ATF4 inhibited STAT3 phosphorylation and that STAT3 phosphorylation was restored by transfection with si-ATF4 and the addition of Colivelin (Fig. 6C). GPX4 expression was downregulated, ACSL4 expression was upregulated after transfection with si-ATF4, GPX4 expression increased, and ACSL4 expression decreased after inhibition of ATF4 expression and the addition of Colivelin (Fig. 6D). An increase in Fe^2+^ content occurred following the inhibition of ATF4 expression and a decrease in Fe^2+^ content occurred following the inhibition of ATF4 expression and the addition of Colivelin (Fig. 6E). ROS levels, detected by flow cytometry, increased after transfection with si-ATF4 and decreased after transfection with si-ATF4 and the addition of Colivelin (Fig. 6F). BODIPY staining showed that transfection with si-ATF4 promoted lipid peroxidation levels, and transfection with si-ATF4 and the addition of Colivelin led to a decrease in lipid peroxidation levels (Fig. 6G). Next, the IC_50_ of sorafenib was measured by CCK-8 and showed that transfection with si-ATF4 significantly decreased the IC_50,_ and transfection with si-ATF4 and the addition of Colivelin increased the IC_50_ (Fig. 6H). These results indicated that transfection with si-ATF4 resulted in increased sensitivity of Huh7 to sorafenib, while transfection with si-ATF4 and the addition of Colivelin decreased sensitivity. Western blot assays showed that the expression of the drug resistance-associated proteins ABCG2 and P-GP in the cells decreased after inhibition of ATF4 expression and increased after inhibition of ATF4 expression and the addition of Colivelin (Fig. 6I). The above results suggest that downregulation of ATF4 inhibits the phosphorylated nuclear translocation of STAT3, promotes ferroptosis, and increases the sensitivity of Huh7 cells to sorafenib, while activation of STAT3 reverses the effect of ATF4 downregulation. Thus, the ATF4/STAT3 pathway mediates cellular ferroptosis to improve sorafenib resistance.

### Metformin mediates ferroptosis and sorafenib resistance via ATF4/STAT3 in vivo

**Fig. 7:**
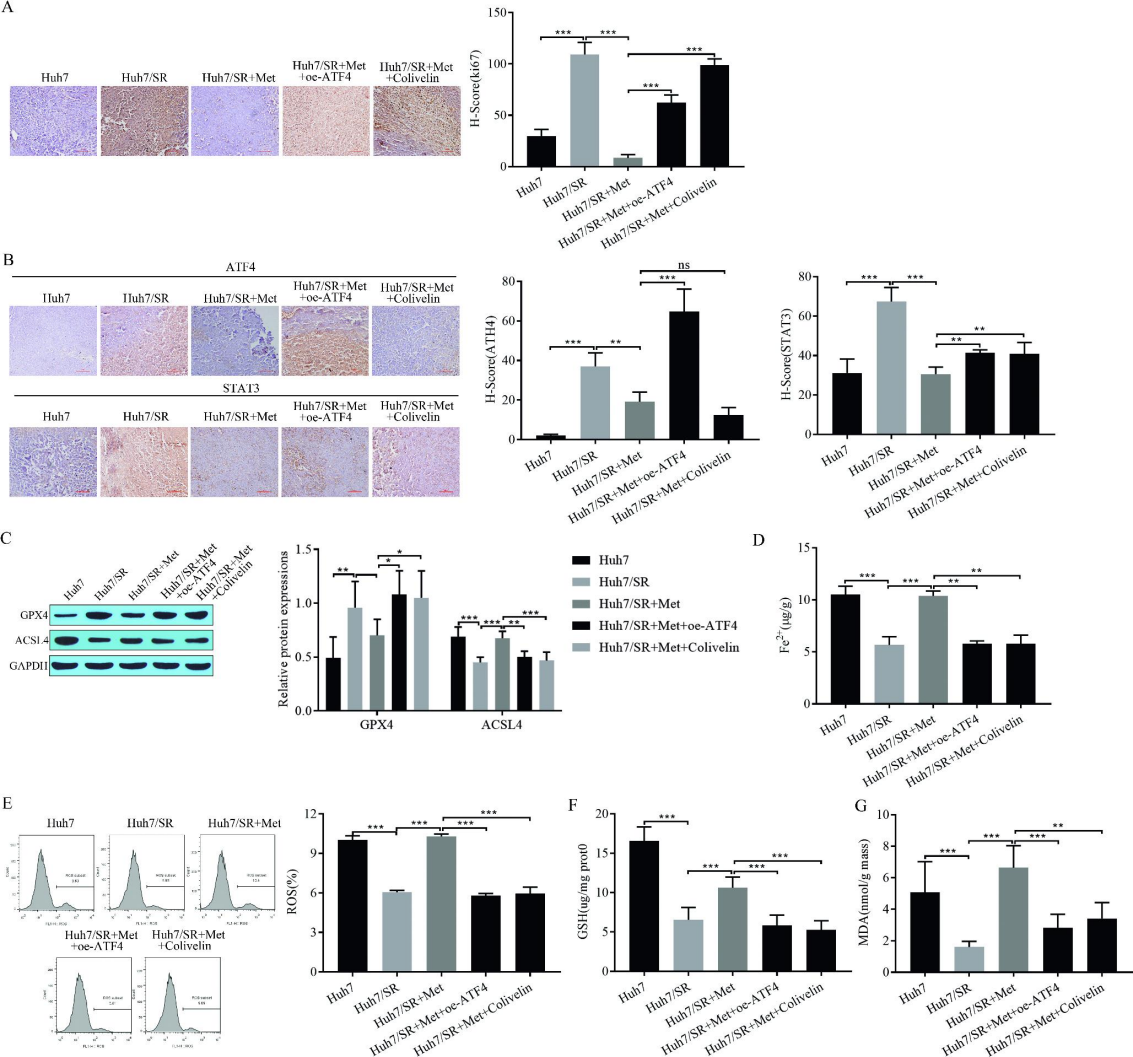
Metformin mediates ferroptosis and sorafenib resistance via ATF4/STAT3 in vivo. **(A)** Ki-67 immunohistochemical staining; **(B)** immunohistochemical detection of ATF4 and STAT3; **(C)** Western blot for expression of ferroptosis-related proteins GPX4 and ACSL4; **(D)** Fe^2+^ content; **(E)** Flow cytometry for ROS; **(F)** expression of MDA and GSH. *P < 0.05, **P < 0.01, ***P < 0.001, ns: no significant difference

At the animal level, we verified that metformin mediates ferroptosis and sorafenib resistance via ATF4/STAT3 by injecting metformin and transfecting oe-ATF4 or adding Colivelin. Immunohistochemical results showed that Ki-67 decreased after metformin injection and increased after metformin injection and transfection with oe-ATF4 or metformin injection and the addition of Colivelin (Fig. 7A). These results indicated that metformin inhibited tumor cell proliferation, while transfection with oe-ATF4 or the addition of Colivelin both reversed the effect of metformin. Immunohistochemistry showed that injection of metformin inhibited the expression of ATF4 and STAT3, while injection of metformin and transfection with oe-ATF4 resulted in increased expression of ATF4 and STAT3 and injection of metformin and the addition of Colivelin significantly inhibited the expression of ATF4 and had no significant effect on the expression of STAT3 (Fig. 7B). Western blot analysis showed that GPX4 decreased and ACSL4 expression increased after metformin injection, and GPX4 increased and ACSL4 expression decreased after metformin injection and oe-ATF4 transfection or metformin injection and Colivelin addition (Fig. 7C). Metformin promoted Fe^2+^ accumulation, whereas transfection with oe-ATF4 or the addition of Colivelin both reversed the effect of metformin (Fig. 7D). ROS levels were significantly increased by flow cytometry after metformin injection, whereas transfection with oe-ATF4 or the addition of Colivelin both reduced ROS production after metformin injection (Fig. 7E). MDA and GSH expression increased after metformin injection, whereas transfection with oe-ATF4 or the addition of Colivelin after metformin injection resulted in a decrease in MDA and GSH expression (Fig. 7F-G). The above results suggest that metformin mediates ferroptosis and sorafenib sensitivity via ATF4/STAT3 in vivo, which in turn inhibits hepatocellular carcinoma progression.

## Discussion

Sorafenib is a first-line chemotherapeutic agent for advanced hepatocellular carcinoma. However, sorafenib resistance is becoming increasingly common and is a major cause of poor patient prognosis and a challenge to clinical care [24]. Ferroptosis has been reported to be strongly associated with sorafenib resistance in hepatocellular carcinoma [25]. A recent study reported that depletion of intracellular iron stores protects HCC cells from the cytotoxicity of sorafenib and prevents sorafenib from inducing oxidative stress in the cells [26]. In addition, sorafenib was found to induce ferroptosis in different cancer cell lines [27]. Sorafenib-induced ferroptosis may be a potent mechanism for the induction of HCC cell death [28]. Therefore, the induction of ferroptosis in hepatocellular carcinoma cells may be an effective way to enhance the anticancer activity of sorafenib. In the present study, we found that metformin promoted ferroptosis and sorafenib sensitivity in hepatocellular carcinoma cells via ATF4/STAT3.

Metformin is currently a first-line drug for the treatment of type 2 diabetes. Studies have shown that metformin can induce cell cycle arrest and promote cell death in hepatocellular carcinoma [29] and thus inhibit the progression of HCC. In Lai et al. [13], metformin was found to restore HCC cell sensitivity to sorafenib via AMPK-dependent autophagy activation. In addition, metformin enhanced sensitivity to sorafenib by reversing the EMT process in HepG2 cells and reducing the formation of cancer stem cells [30]. Consistent with these studies, we found that metformin reduced the IC_50_ of sorafenib in hepatocellular carcinoma cells, inhibited resistance in sorafenib-resistant cells, and mitigated cell migration and invasion to reverse sorafenib resistance in hepatocellular carcinoma cells. In earlier studies, metformin was also found to induce ferroptosis in an AMPK-independent manner to inhibit tumor growth [16, 31]. Here, we demonstrated that metformin promotes Fe^2+^ and lipid ROS levels and promotes ferroptosis in HCC cells. Using the ferroptosis inhibitor Fer-1, we found that the effect of metformin on Huh7 sorafenib resistance was attenuated, suggesting that metformin may alleviate HCC sorafenib resistance by promoting ferroptosis.

Metformin activation of ATF4 induces inhibition of the mitochondrial respiratory chain, which in turn affects tumor growth [32, 33]. There is growing evidence that ATF4 promotes the transcription of genes that support respiration and alleviate mitochondrial stress [34, 35]. In the present study, we found that upregulation of ATF4 reversed the effects of metformin, inhibited ROS production, reduced Fe^2+^ and lipid peroxidation levels, and inhibited ferroptosis in HCC cells. In addition, STAT3, a transcription factor important for HCC development [36], promotes hepatocellular carcinoma stem cell expansion and has important functions in tumorigenesis, progression, recurrence, and drug resistance in hepatocellular carcinoma [37, 38]. We found that ATF4 was expressed in the nucleus and STAT3 was expressed in the cytoplasm, indicating that the regulatory interaction between ATF4 and STAT3 did not occur at the transcriptional level; however, the immunoprecipitation results showed a protein interaction between ATF4 and STAT3. Previous studies have reported increased nuclear translocation of activated STAT3 in the mammary gland of transgenic ATF4 mice with upregulated expression of their target genes [39]. Using immunoblot analysis, we found that STAT3 undergoes phosphorylated nuclear translocation. In addition, using ZDOCK 3.0.2 for binding force raw letter prediction, we obtained the protein interaction mode of ATF4 with STAT3. We concluded that metformin mediates sorafenib sensitivity and ferroptosis via ATF4/STAT3.

In conclusion, our study demonstrates that metformin promotes ferroptosis and sorafenib sensitivity in hepatocellular carcinoma cells via ATF4/STAT3. As metformin is a mitochondrial complex enzyme inhibitor and hepatocellular carcinoma cells have higher mitochondrial respiration efficiency [40], they will be more sensitive to treatment for HCC. Studies have shown few complications when metformin is given to cancer patients, including hepatocellular carcinoma patients, and although studies have suggested that metformin may impair cognitive function, a causal relationship between metformin prescription and cognitive impairment has not been confirmed. In conclusion, metformin is a safe drug for cancer patients [41]. The use of metformin may enhance the therapeutic efficacy of sorafenib in hepatocellular carcinoma. However, long-term exposure to metformin has been found to lead to metabolic adaptation and drug resistance in HCC cells, and high doses of metformin can also lead to lactic acidosis [42, 43]. At the same time, ATF4 and STAT3 inhibitors may have further applications in sorafenib-resistant liver cancer patients, and understanding the role of ATF4/STAT3 molecular pathways in HCC progression will contribute to the development of potential drugs for targeted treatment of liver cancer.

## Electronic supplementary material

Below is the link to the electronic supplementary material.

Supplementary Material 1

## Data Availability

The datasets used and/or analyzed during the current study are available from the corresponding author upon reasonable request.
