# Supplementary material for: Metformin promotes ferroptosis and sensitivity to sorafenib in hepatocellular carcinoma cells via ATF4/STAT3
Source: Mol Biol Rep. 2023 Jun 16;50(8):6399–413. doi: 10.1007/s11033-023-08492-4 (PMC10374833; doi:10.1007/s11033-023-08492-4)
Supplement: Supplementary file 1 — Supplementary Material 1 [file 11033_2023_8492_MOESM1_ESM.pdf]

Editing Certificate

This document certifies that the manuscript

Metformin promotes ferroptosis and sensitivity to sorafenib in hepatocellular carcinoma cells via ATF4/STAT3

prepared by the authors

Zongqiang Hu1, Yingpeng Zhao1, Laibang Li1, Jie Jiang1, Wang Li1, Yuanyi Mang 1, Yang Gao1, Yun Dong1, Jiashun Zhu1, Chaomin Yang1, Jianghua Ran1*, Li Li1*, Shengning Zhang1*

was edited for proper English language, grammar, punctuation, spelling, and overall style by one or more of the highly qualified native English speaking editors at AJE.

This certificate was issued on **April 14, 2023** and may be verified on the AJE website using the verification code **5B19-BCDD-FCBC-3BBB-25A1**.

Neither the research content nor the authors' intentions were altered in any way during the editing process. Documents receiving this certification should be English-ready for publication; however, the author has the ability to accept or reject our suggestions and changes. To verify the final AJE edited version, please visit our verification page at aje.com/certificate. If you have any questions or concerns about this edited document, please contact AJE at support@aje.com.

AJE provides a range of editing, translation, and manuscript services for researchers and publishers around the world. For more information about our company, services, and partner discounts, please visit aje.com.
